# User needs and design opportunities for a conversational agent for tuberculosis treatment: A mixed-methods study

**DOI:** 10.1371/journal.pdig.0001720

**Published:** 2026-09-22

**Authors:** Joon Sang Baek, Sehwa Choi, Seojin Sung, Vasuki Rajaguru, Youngmok Park

**Affiliations:** 1 Department of Integrated Design, Yonsei University, Seoul, South Korea; 2 Department of Integrated Design, Tsinghua University, Beijing, China; 3 Department of Convergence Design, Hannam University, Daejeon, South Korea; 4 Department of Healthcare Management, Graduate School of Public Health, Yonsei University, Seoul, South Korea; 5 Institute for Innovation in Digital Healthcare, Yonsei University, Seoul, South Korea; 6 Division of Pulmonary and Critical Care Medicine, Department of Internal Medicine, Yonsei University College of Medicine, Severance Hospital, Seoul, South Korea; University of Washington, UNITED STATES OF AMERICA

## Abstract

Tuberculosis (TB) remains a major global health challenge requiring prolonged treatment that is often complicated by adverse drug reactions (ADRs), stigma, and poor treatment adherence. Although digital health interventions show promise in supporting TB care, patients’ needs, expectations, and design requirements for conversational agents remain poorly understood. This study identified challenges experienced by healthcare professionals and patients with TB and explored opportunities for designing a user-centered conversational agent for TB care. A mixed-methods approach was adopted, comprising surveys of 107 healthcare professionals and 31 patients with TB, followed by in-depth interviews with 10 patients. The survey assessed treatment challenges, adherence barriers, and digital health needs. The interview data explored participants’ treatment experiences and expectations for conversational agent–based support. Quantitative data were analyzed using descriptive statistics, and qualitative data were analyzed using inductive thematic analysis. Healthcare professionals reported ADR (25.1%), multidrug-resistant TB (17.1%), drug interaction management (15.6%), and low treatment adherence (15.6%) as key challenges. Patients’ needs focused on ADR consultation (35%), TB information (21.7%), and diagnostics (20%). The majority wanted to use mHealth (62.1%), with specific interests in side effect guidance (20.4%), self-diagnosis (13%), and treatment information (12.5%). The interviews revealed patients’ multifaceted needs related to TB treatment, including consistent communication with healthcare providers, effective ADR management, accurate and accessible TB information, social and psychological support during treatment, usable and accessible digital health solutions, and affordable services. These findings identified key design opportunities, including TB awareness, timely and personalized information, treatment adherence support, transparent treatment progress, psychosocial support, accessible digital services, and affordability. Healthcare professionals and patients reported unmet needs that conversational agents could potentially address. These findings provide user-centered guidance for developing conversational agents and contribute to evidence on digital health interventions for TB treatment support.

## Author summary

Tuberculosis (TB) treatment can be difficult to complete because it is prolonged and often involves side effects, uncertainty, stigma, and limited opportunities to ask questions during clinic visits. Digital tools such as conversational agents can help patients manage treatment; however, few studies have examined what patients and healthcare professionals need from such tools in TB care. In this mixed-methods study, we surveyed 107 healthcare professionals and 31 patients with TB and interviewed 10 patients in South Korea to identify barriers to use, unmet needs, and design priorities for a user-centered conversational agent supporting TB care (hereafter, anti-TB conversational agent). Healthcare professionals highlighted adverse drug reactions (ADRs), adherence-related challenges, multidrug-resistant TB, and drug interaction management as major concerns. Patients emphasized the need for rapid guidance regarding ADRs, clearer TB information, easier communication with providers, and emotional support during treatment. Patients preferred solutions that were user-friendly, affordable, and integrated into familiar platforms. Accordingly, we identified key design priorities, including early detection and TB awareness, timely and customized information access, personalized support for treatment adherence, transparency in treatment progress, psychosocial and technological support, and treatment affordability. These findings can guide the development and future evaluation of user-centered conversational agents for TB treatment-adherence support.

## Introduction

Tuberculosis (TB) remains one of the top 10 causes of death worldwide, with approximately 10.6 million new cases reported in 2021 [1]. In South Korea, TB continues to be an important health concern, with an incidence rate of 59 cases per 100,000 people. Additionally, the country has the second-highest TB mortality rate among all Organisation for Economic Co-Operation and Development countries, at approximately four deaths per 100,000 people [1,2].

The World Health Organization’s (WHO) Global TB Program identified a patient-centered approach as a key pillar supporting the End TB Strategy [3]. A patient-centered approach is important in TB care and control, given the need for universal access to care and support that is based on individual needs and experiences. This approach empowers patients as active participants in their TB care and involves control strategies such as case reports and peer support. Further, this approach improves the quality of care by establishing mutual trust in the patient–healthcare provider relationship [4]. Digital technologies have proven effective in providing patient-centered TB care that is both accessible and tailored to individual patients’ needs. One example is digital adherence technology, aimed at supporting and monitoring medication intake to improve treatment adherence.

Digital adherence technologies, including SMS-based tools, video-observed therapy (VOT), and medication event-monitoring systems, have been shown to improve TB treatment adherence and disease control [3,5,6]. Similarly, mobile health (mHealth) tools such as monitoring systems, reminder applications, and connected mobile devices have emerged as effective approaches to support adherence in high-burden settings [7–9]. Conversational agents, including chatbots and virtual health assistants, further support treatment by facilitating communication between patients and healthcare providers while improving information access, health literacy, and patient outcomes [10–12]. These tools are scalable, available 24/7, and can reduce the healthcare workload by delivering timely, personalized supportive care [13].

Although digital health technologies have become increasingly common in TB care, existing studies on designing conversational agents to address the multifaceted needs of patients and healthcare professionals remain limited [14–18]. Previous research highlights that when developing culturally sensitive conversational agents, understanding users’ needs and challenges is important to ensure their relevance and effectiveness across diverse patient populations worldwide [19]. Despite the growing evidence supporting digital adherence technologies and mHealth interventions for TB care, research on conversational agents specifically designed for TB treatment remains limited. Most studies have focused on SMS reminders, VOT, medication monitoring systems, or evaluations of digital adherence outcomes, rather than on understanding end users’ needs, preferences, and experiences during the design phase [15,16,18,20]. The lack of empirical knowledge on this topic hinders the design of user-centered conversational agents and presents barriers to their adoption [21–24], making it challenging to integrate these agents into conventional clinical practice.

Thus, important gaps remain in our understanding of how conversational agents should be designed from a user-centered perspective and implemented to support TB treatment. To address this issue, we developed a rule-based anti-TB chatbot in 2022 that provides TB education, medication reminders, appointment reminders, and adverse drug reaction (ADR) reporting support.

We conducted a pilot study involving patients with TB and healthcare professionals to explore their experiences, needs, and expectations regarding conversational agent-supported care. These findings will inform the development of user-centered conversational agents for TB treatment. Specifically, this study asked the following questions: (1) What challenges in TB treatment could conversational agents help address? (2) What needs and expectations do patients, and healthcare professionals have for such tools? and (3) Which design considerations should guide the development of conversational agents for TB care? To our knowledge, this is one of the first mixed-methods studies to systematically examine both patients with TB and healthcare professionals’ needs, expectations, and design preferences for a conversational agent integrated into routine TB treatment support.

## Materials and methods

### Anti-TB chatbot design

An anti-TB chatbot prototype was developed on an open-source platform and integrated into KakaoTalk, a messaging application widely used in South Korea. Using a rule-based conversational system with predefined menus and responses, the chatbot provided three main functions: (1) TB education and information on treatment, hospitals, and support services; (2) medication reminders and daily dosing confirmation; and (3) ADR reporting and monitoring (S1 Fig).

**Fig 1:**
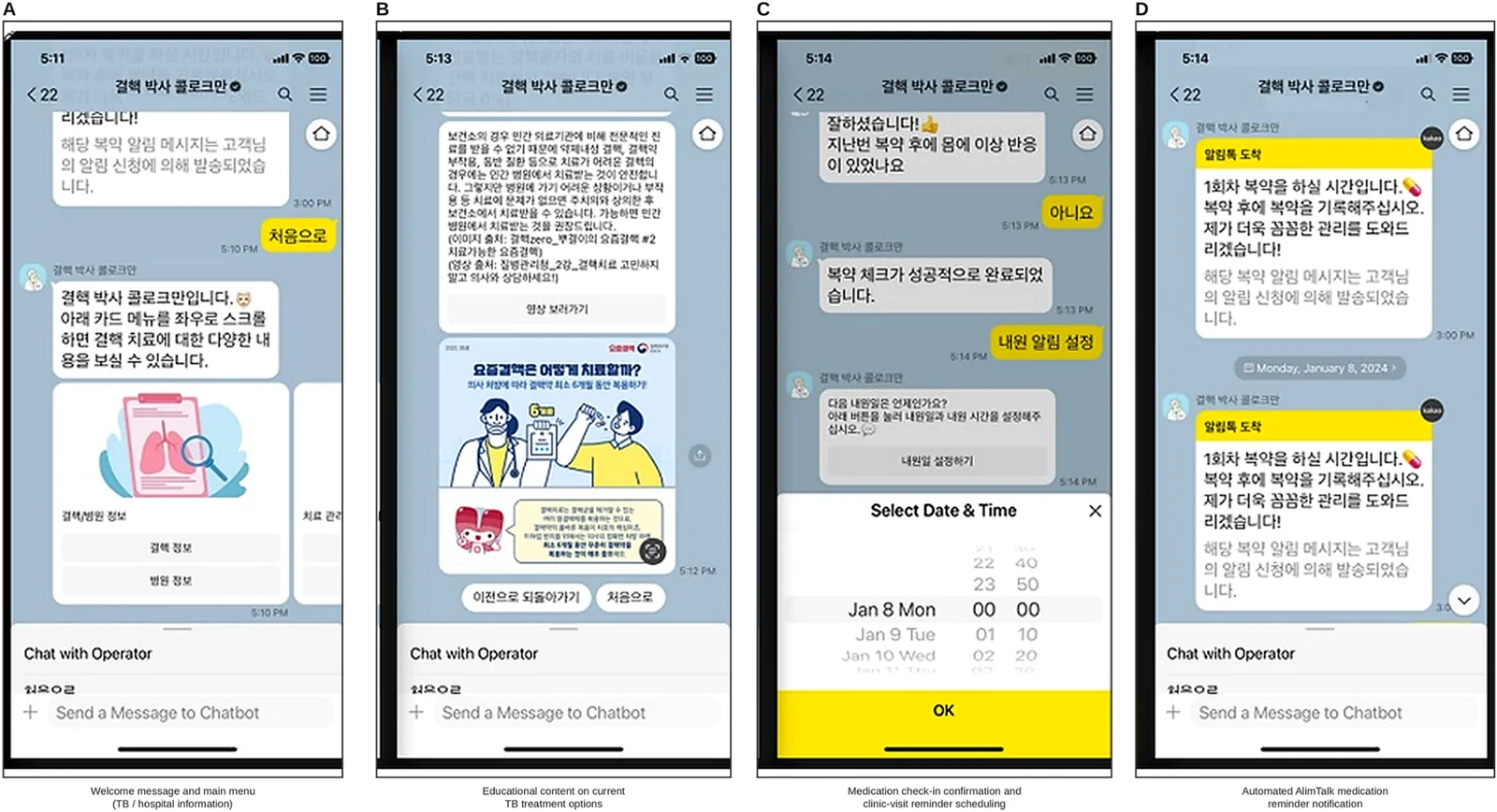
Anti-TB chatbot screenshots illustrating the user interface and key functions of the TB chatbot in the KakaoTalk messaging platform. The chatbot supports TB patients with treatment education, symptom monitoring, and medication reminders. Screens show (1) welcome message and information menu, (2) educational content: TB-related knowledge in a visual and accessible format, (3) chatbot checks for ADRs post-medication and medication alarm set-up, and (4) scheduled reminder for medication intake. The chatbot supports TB treatment adherence by offering reminders, educational support, and real-time two-way communication.

The chatbot content was developed from national TB guidelines, WHO resources, and patient education materials, and was reviewed by TB specialists to ensure its accuracy and readability. The chatbot was designed to support not replace communication with healthcare professionals and did not provide diagnoses or treatment recommendations. Minimal user data were collected, and all information was managed in accordance with institutional privacy requirements and the Personal Information Protection Act of South Korea. An overview of the chatbot’s architecture, content governance, and privacy framework is shown in Fig 1. Representative user interface screenshots are provided with the original Korean content translated into English for international readers (S2 Fig).

### Study design and participants

A mixed-method approach was used to explore user needs and inform the design of conversational agents. Surveys were conducted with healthcare professionals and patients with TB, followed by in-depth interviews with patients to gain a deeper understanding of their treatment experiences and support needs. All participants provided their written informed consent. Patients who participated in both the survey and interview were awarded a USD-50 gift voucher, and healthcare professionals who completed the survey received a USD-10 gift coupon.

Participants were recruited from tertiary referral hospitals, general hospitals, clinics, and public health centers involved in TB care. Eligible participants included physicians and nurses engaged in TB diagnosis, treatment, counseling, or case management. Eligible participants were adults receiving TB treatment at Severance Hospital who had taken anti-TB medication for at least three months and were able to provide informed consent. Patients with severe cognitive or communication difficulties that precluded participation were excluded. This study was designed as an exploratory, formative, needs-assessment study; therefore, no formal sample size calculation was performed.

### Measures

The 23-item questionnaire included multiple-choice, Likert-scale, and open-ended questions on demographics, TB treatment challenges, directly observed therapy (DOT)/VOT use and its effectiveness, digital health needs, and feedback on the anti-TB chatbot prototype. The questionnaire included items adapted from the unified theory of acceptance and use of technology [25, 26] and covered five domains: performance expectancy, effort expectancy, social influence, facilitating conditions, and behavioral intention. The internal consistency of the Likert-scale items was good (Cronbach’s alpha = 0.84).

A separate 24-item survey was carried out to 31 patients with TB receiving treatment at the study hospital. The survey was developed based on a review of the literature and input from TB specialists, digital health researchers, and user-centered design experts [26,27]. The questionnaire explored patients’ experiences throughout the TB treatment journey, including prior TB education, treatment-related challenges, ADRs, sources of treatment information, and support needs during diagnosis and treatment. Moreover, patients were asked about their actions in response to ADRs, preferences for digital health support, desired features of a smartphone-based TB support application, willingness to use such technologies, and acceptable subscription costs.

### Data collection

Data was collected using two surveys and follow-up interviews. First, an online survey of healthcare professionals was conducted from May to July 2022. The questionnaire was distributed to an online community of physicians and nurses involved in TB care in tertiary hospitals, general hospitals, clinics, and public health centers. Convenience sampling was used because TB clinicians in South Korea have a small and dispersed workforce concentrated in public–private mix hospitals and public health centers; Of the 210 healthcare professionals invited to participate, 107 completed the survey, yielding a response rate of 50.9%. Second, a paper-based survey was administered to 31 patients who had received TB treatment at the corresponding author’s institution during the same period. Patients were approached consecutively if they were aged 18 years or older, had a confirmed diagnosis of TB, had been receiving anti-TB treatment for at least three months, and were able to communicate in Korean. Patients with severe illness, cognitive impairment, or other conditions that limited their ability to participate in the study were excluded. Questionnaires were completed during routine hospital visits, and responses were entered into a digital database by the research team. Finally, semi-structured interviews were conducted with 10 patients who participated in the survey from July to September 2022. To capture the diversity of their experiences, interviewees were purposively recruited based on their gender, age, cohabitation type, and family support level (S1 Table). This sample size was within the recommended range (6–12) for non-probability samples to reach saturation in a reasonably homogeneous group of patients undergoing TB treatment at a single hospital who share similar ethnic and cultural characteristics [28]. The questionnaire included questions on how the patients discovered that they had TB, challenges during TB treatment, why TB treatment needed specific support, and reasons for seeking specific features in the digital solution. The interviews lasted approximately 30 minutes, and patient responses were recorded for transcription. All patients provided written informed consent and were permitted to withdraw from the study at any time.

### Data analysis

Quantitative data were analyzed using SPSS software, version 26 (IBM Corp., Armonk, NY, USA). Descriptive statistics were used for quantitative data analysis, including frequency (%) for all categorical variables and means (standard deviations) for continuous variables. Responses to Likert-scale items were summarized using mean scores to assess participants’ perceptions of the chatbot’s usefulness, ease of use, social influence, facilitating conditions, and behavioral intentions. Item-level analyses were performed to identify the aspects of the chatbot that were viewed positively and areas requiring improvement.

Qualitative data from open-ended survey responses and interviews were analyzed using inductive content analysis with NVivo™ software (Release 1.7) [29]. Reporting of the qualitative component was guided by the Consolidated Criteria for Reporting Qualitative Research (COREQ). Verbatim transcripts were divided into units of meaning. The extracted meaning units were categorized into codes, which were further categorized into themes. Consensus coding was conducted to ensure inter-coder reliability. Two researchers trained in qualitative data analysis independently coded a subset of the dataset (32.3%). Through negotiated agreement, a final consensus of 90% was reached. The remaining 10% of irreconcilable segments were marked as “undetermined” and excluded from the results [30].

### Ethics statement

This study was approved by the Institutional Review Board of Yonsei University Health System, Severance Hospital, for the healthcare-provider survey (IRB No. 4-2022-0407) and the patient survey with in-depth interviews (IRB No. 4-2022-0164). All participants provided informed consent, and the healthcare provider survey qualified for a waiver of written consent. The studies were reviewed using expedited procedures and conducted in accordance with the Declaration of Helsinki and applicable national regulations.

## Results

### Healthcare professionals’ characteristics

Among the 107 healthcare professionals surveyed, 69.2% were women, and the majority were aged 40–49 years (42.9%). Most worked in general (52.3%) or tertiary hospitals (43%) as internal medicine physicians (54.2%) or public–private mix nurses (43.9%). Institutions ranging from small clinics to large hospitals treat over 300 TB patients annually (Table 1).

**Table 1:** General characteristics of surveyed healthcare professionals (HCP) (n = 107).

Characteristics		n (%)
**Gender**	Men	33 (30.8)
	Women	74 (69.2)
**Age (years)**	≤ 39	35 (32.7)
	40–49	46 (43)
	50–59	22 (20.6)
	≥ 60	4 (3.7)
		
**Type of medical institution**	General hospital	56 (52.3)
	Tertiary hospital	46 (43)
	Clinic	1 (0.9)
	Others	4 (3.7)
**Job (specialization)**	Internal medicine physician	58 (54.2)
	General physician	1 (0.9)
	Public–private mix nurse	47 (43.9)
	Nurse	1 (0.9)
**Annual number of patients with TB treated by the institution**	< 10	1 (0.9)
	11–50	10 (9.3)
	51–100	22 (20.6)
	101–300	50 (46.7)
	≥ 301	24 (22.4)
**Annual number of patients with TB treated by the respondent**	< 10	16 (14.9)
	11–50	33 (30.8)
	51–100	13 (12.1)
	101–300	5 (4.7)
	≥ 301	3 (2.8)
	No response	37 (34.6)

n, frequency; TB, tuberculosis.

### Challenges in TB treatment and digital health technology needs among healthcare professionals

The main challenges in TB treatment were ADR management (25.1%), multidrug-resistant TB (17.1%), drug-interaction verification (15.6%), and poor adherence (15.6%). Barriers to adherence included ADRs (43%), lack of self-management (42.1%), and insufficient social support (8.4%). Most respondents (76.6%) did not use DOT or VOT. Healthcare professionals expressed a need for technological solutions such as simplified medical record access (26.2%), rapid diagnostic tools (24.3%), and integration with mobile and wearable devices (21.5%) (S1 Table).

### Patients’ characteristics

More than half of the surveyed patients were men (61.3%). Furthermore, they were mostly South Korean nationals (96.8%) aged 40 and above (93.5%). Moreover, most lived with family (93.5%) and received a high mean family support score (6.8 ± 1.4 out of 10). Medication adherence was high (9.87 ± 0.34 out of 10). The general characteristics of patients with TB are presented in Table 2 and Table 3 presents those of interview participants.

**Table 2:** General characteristics of surveyed patients (n = 31).

Characteristics		n (%)
**Gender**	Men	19 (61.3)
	Women	12 (38.7)
**Nationality**	Korean	30 (96.8)
	Chinese	1 (3.2)
**Age (years)**	30–39	2 (6.5)
	40–49	7 (22.6)
	50–59	5 (16.1)
	60–69	9 (29.0)
	≥ 70	8 (25.8)
**Marital status**	**Married**	28 (90.3)
	Single	3 (9.7)
**Education**	Elementary school and below	4 (12.9)
	Middle school	1 (3.2)
	High school	11 (35.5)
	University	13 (41.9)
	Graduate school or above	2 (6.5)
**Occupation**	**Specialist**	8 (25.8)
	**Unemployed**	7 (22.6)
	**Housewife**	7 (22.6)
	**Office worker**	5 (16.1)
	Others*	4 (12.8)
**Monthly income**	≥ 5000K KRW or 5000 USD	6(19.4)
	3000K–5000K KRW or 3000–5000 USD	9(29.0)
	1000K–3000K KRW or 1000–3000 USD	6(19.4)
	<1000K KRW or <1000 USD	10(32.3)
**Economic burden of** TB **treatment**	**It is not a burden**	30 (96.8)
	**It is not too much of a burden**	1 (3.2)
**Alcohol consumption**	Never	15 (48.4)
	Former	10 (32.3)
	Current	6 (19.4)
**Smoking**	Never	15 (48.4)
	Former	14 (45.2)
	Current	2 (6.5)
**Cohabitation status**	Joint	29 (93.5)
	Single	2 (6.5)
**Cohabitant (multiple responses, n = 43)**	Spouse	24 (55.8)
	Children	15 (34.9)
	Parents	2 (4.7)
	Siblings	1 (2.3)
	Others (friend)	1 (2.3)
**Response to TB treatment (M ± SD)**	Level of family support	6.8 (1.4)
	Medication adherence	9.9 (4.5)

N, frequency; SD, standard deviation; *others, unskilled labor, self-employed, and agricultural workers.

**Table 3:** Information on interviewed patients with tuberculosis (n = 10).

ID	Gender	Age (years)	Marital status	Alcohol	Smoking	Family support (%)	Adherence (%)
1	M	60–69	Joint	Present	Former	90	100
4	F	30–39	Joint	Never	Never	90	100
5	F	40–49	Joint	Former	Never	0	100
8	F	60–69	Single	Never	Never	0	100
11	M	60–69	Joint	Present	Present	90	100
13	M	50–59	Joint	Never	Former	100	100
14	M	60–69	Joint	Former	Never	100	100
20	F	40–49	Joint	Never	Never	50	90
25	M	50–59	Single	Present	Present	0	100
31	F	≥70	Joint	Never	Never	100	100

### Patients’ treatment experiences and preferences for digital health technologies

**Fig 2:**
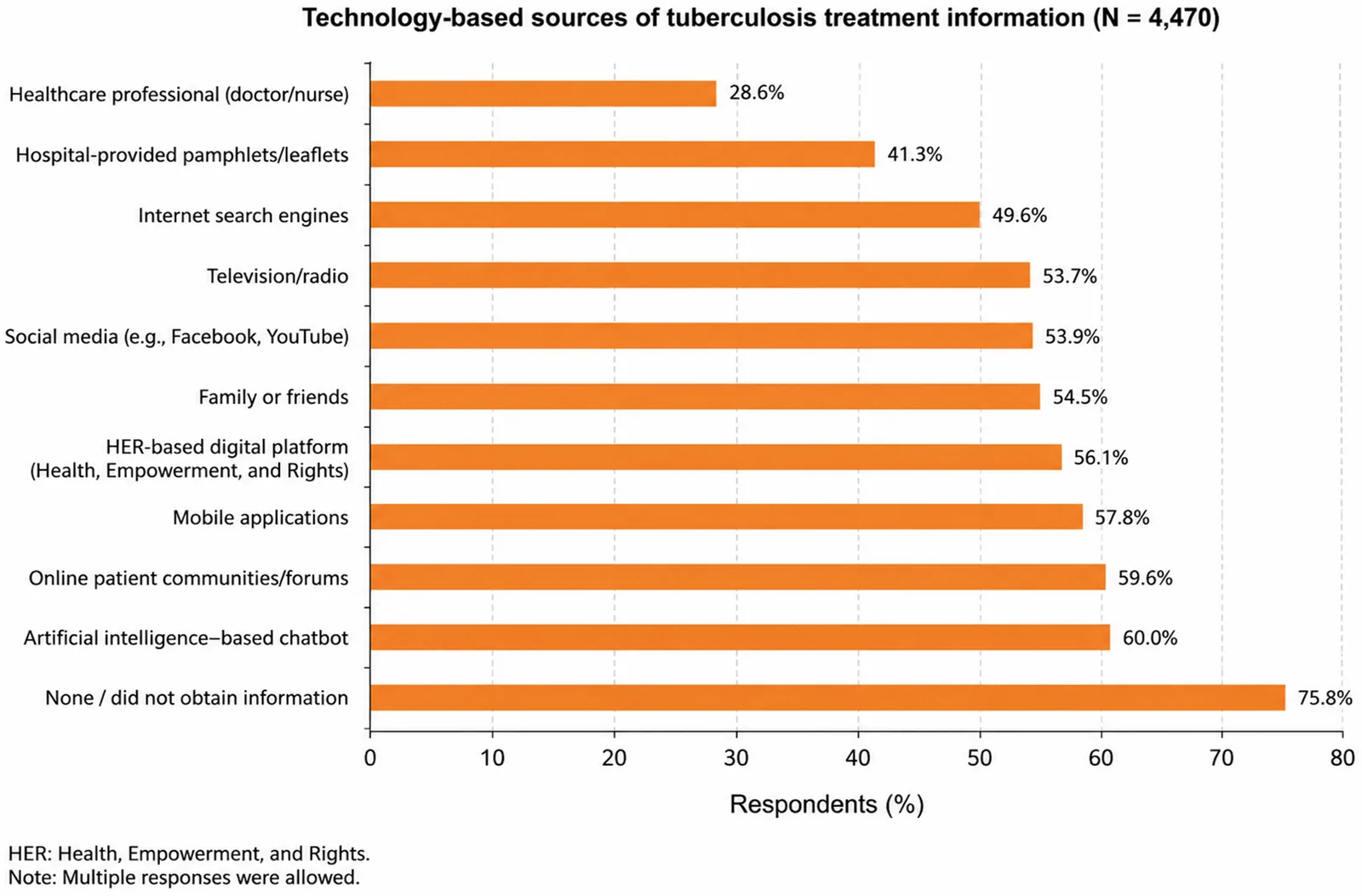
Tuberculosis information and pathways to barriers to treatment adherence experienced by the users.

**Fig 3:**
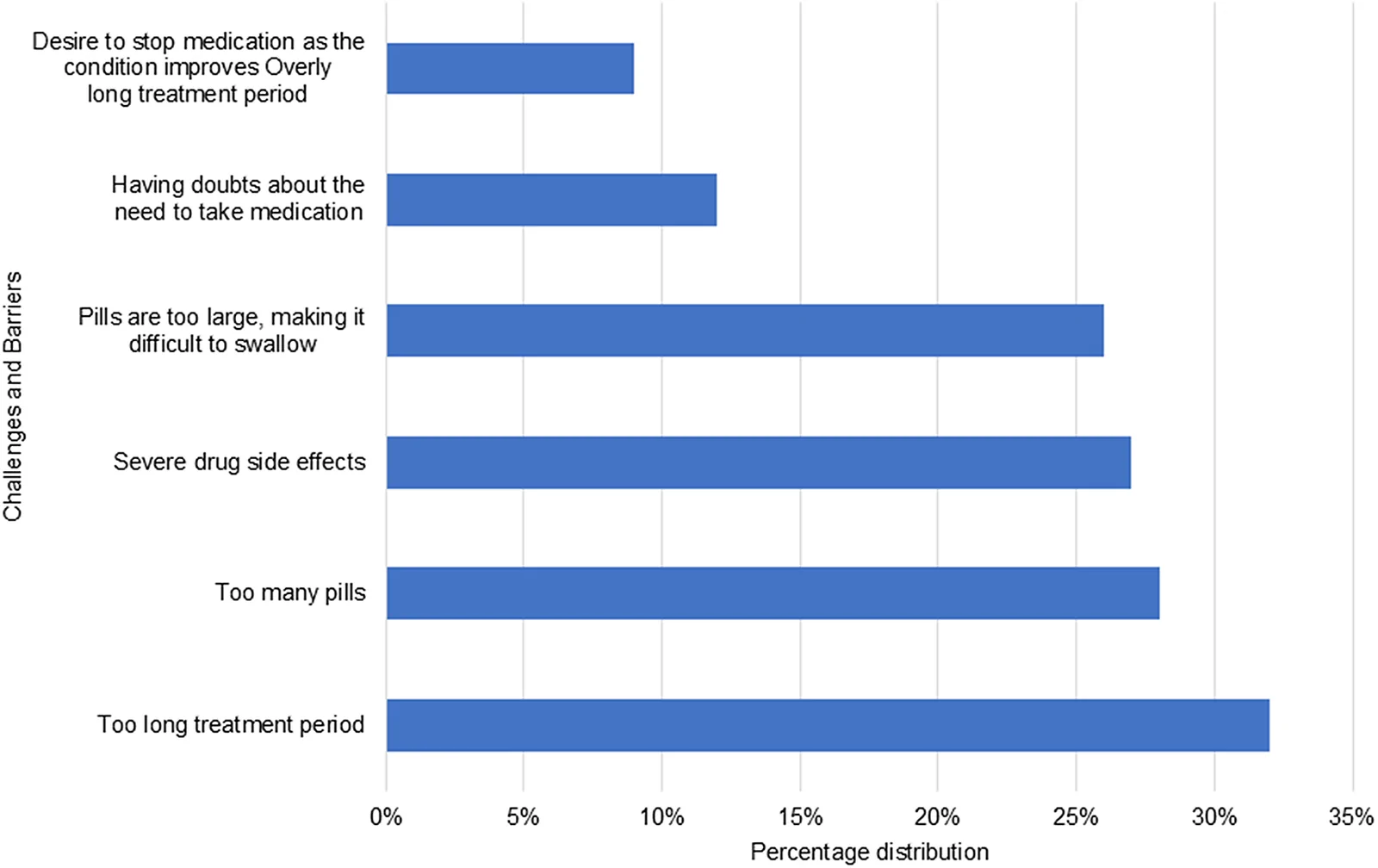
Technology-based tuberculosis information for tuberculosis treatment.

The patients were diagnosed with TB at various healthcare levels and received the most education during treatment. Nearly all patients (n = 30) experienced side effects: 40% sought immediate care, 27.5% waited passively, and 12.5% sought help. The key information sources included doctors (55.3%), the Internet (25.5%), and hospital materials (12.8%). Patients’ needs focused on ADR consultation (35%), TB information (21.7%), and diagnostics (20%). The preferred mHealth tools were side-effect guidance (20.4%), self-diagnosis (13%), and treatment information (12.5%). Most (62.1%) were willing to use mHealth, especially if it was free (93.1%). The details are presented in Figs 2 and 3 and S2 Table.

### Patient needs related to TB treatment

**Fig 4:**
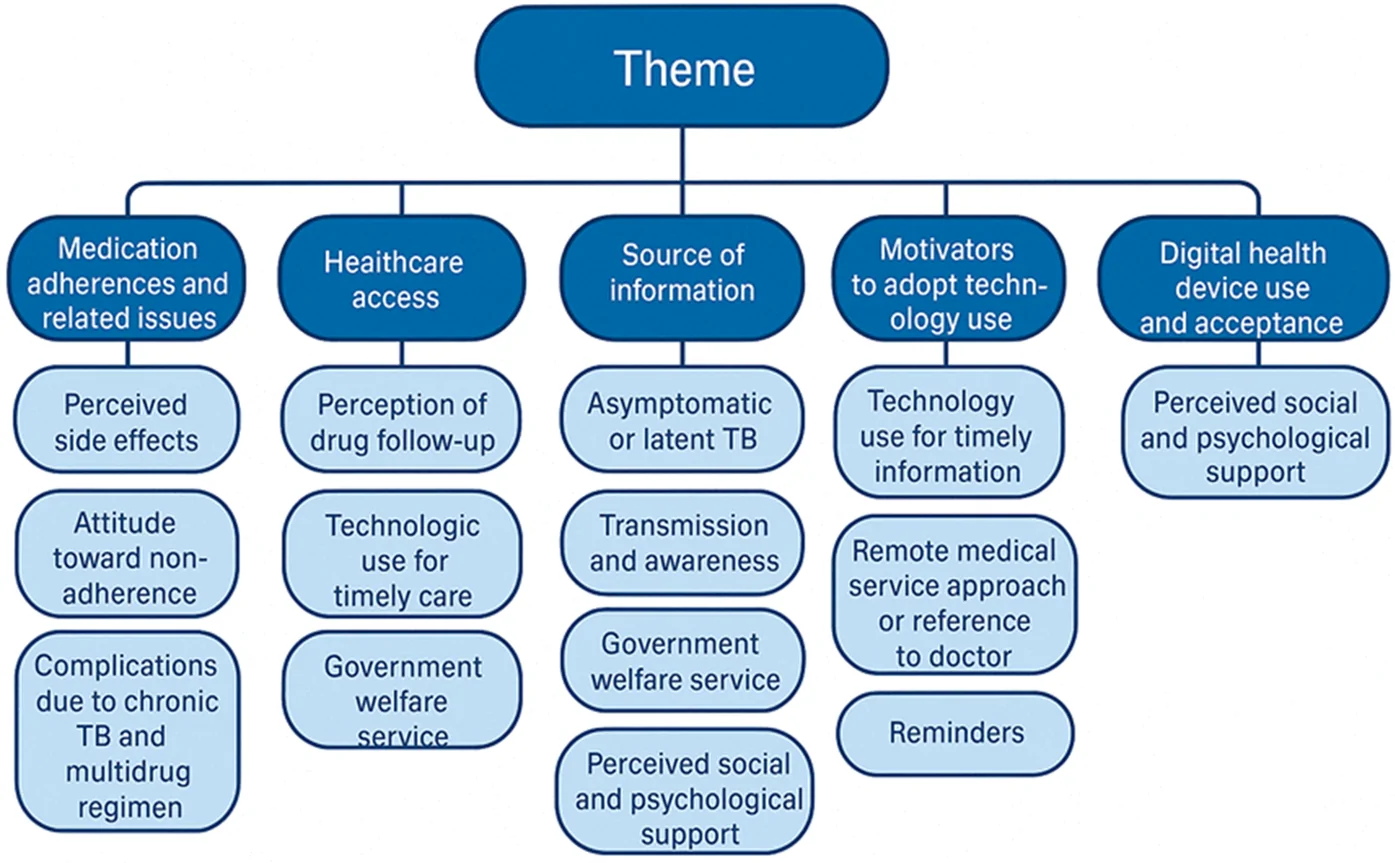
Thematic diagram showing Theme and subthemes derived by the experience of Tuberculosis patients.

Interviews identified six major patient needs: accurate and accessible TB and treatment information, ADR management, timely communication with medical staff, social and psychological support, usable digital health solutions, and affordable services. The thematic results are presented in Fig 4 and Table 4.

**Table 4:** Themes, subthemes, and responses from interviewed patients.

Theme	Subtheme	Patients’ responses
Medication adherence and related issues	Perceived side effects	Patients reported fatigue, nausea, skin rash, cough, hemoptysis, pain, and fear of repeated side effects.
	Perception of drug follow-up	Dose adjustment and prolonged treatment were experienced as burdensome, especially when side effects persisted.
	Attitude toward non-adherence	Long treatment duration, many pills, fasting requirements, forgetfulness, and stress affected medication timing.
	Complications due to chronic TB and multidrug regimen	Patients described hepatotoxicity, hospitalization, comorbid medication burden, and fear of serious complications.
	Healthcare access	Limited clinic hours, unavailable weekend counseling, and busy providers reduced timely support.
Source of information	Asymptomatic or latent TB	Patients wanted clearer information on latent TB, asymptomatic disease, and family testing.
	Transmission and awareness	Patients were uncertain about how they became infected or transmitted TB and wanted self-diagnosis guidance.
	Government welfare services	Patients wanted clearer guidance on medication cost support and public assistance.
Motivators to adopt technology use	Technology use for timely information	Patients wanted easily accessible information on TB, symptoms, medications, and side effects.
	Remote medical service or doctor reference	Patients valued digital support when doctors or counseling centers were unavailable.
	Reminders	Patients wanted reminders for medication and healthcare tasks, including caregiver alerts.
Digital health device use and acceptance	Willingness to pay	Most preferred free services, consistent with free TB treatment, but some accepted low-cost services.
Perceived social and psychological support	Stigma, isolation, and family support	Patients reported stigma, fear of transmission, isolation, and reliance on family support during treatment.

### Accurate and accessible TB information

The patients reported feeling confused about TB, particularly in its asymptomatic or latent forms, and often accepted treatment without fully understanding its effectiveness, duration, or side effects. Limited physician access, unreliable online sources, and complex terminology hindered reliable information seeking, highlighting the need for clear patient-centered information on TB, treatment, specialist care, and support services (S3 Table).

### Effective ADR management

ADRs substantially affect daily life and well-being, causing fatigue, appetite loss, persistent cough, hemoptysis, skin reactions, and sensory changes. Side-effect management was especially difficult among patients with comorbidities because of concerns about drug interactions and liver toxicity. Further, the long treatment duration, complex regimens, and forgetfulness challenged adherence.

### Consistent communication with healthcare providers

Patients reported limited opportunities for detailed discussions with doctors, despite the need for prompt advice on treatment issues. ADRs increased the demand for responsive communication, especially when symptoms were severe. Limited counseling hours prompted interest in searchable after-hours support and 24-hour counseling.

### Social and psychological support during treatment

TB affected patients beyond physical symptoms. Family support helped with daily activities and emotional encouragement, while stigma, transmission fears, and concerns about judgment contributed to isolation and distress, despite reassurance about reduced infectiousness after treatment initiation.

### Usable and accessible digital health solutions

Usability is critical because most patients are older than 60 years [28]. Patients preferred integration with familiar social network services such as KakaoTalk for convenience and comfort, including receiving information and reporting medication intake. For DOT, simple methods, such as text messaging, were favored, although photo confirmation was acceptable.

### Affordable services

Participants strongly preferred free TB-related services, consistent with the free TB treatment offered in South Korea. Some noted that fees reduced accessibility and uptake, although paid models were acceptable if they offered expert guidance comparable to that offered by doctors or other healthcare professionals. The suggested monthly subscription prices ranged from KRW 1,000/USD 1 for public services to KRW 3,000/USD 3 for private services.

## Discussion

### Design opportunities

This mixed-methods study investigated the experiences of TB healthcare professionals and patients and their expectations regarding the use of an anti-TB chatbot to support treatment adherence. The findings identified several design opportunities for conversational agents that support TB treatment.

### Early detection and TB awareness

The analysis showed that patients were diagnosed through various healthcare pathways, often with limited awareness of asymptomatic or latent TB. Consistent with previous studies, this finding highlights the need for public education to improve TB awareness and reduce transmission [16,31,32].

- Design opportunities: Accessible educational content on TB and latent infection, symptom screening guidance, and tools that allow users to share reliable information with family and community members*.*

### Timely and customized information access

After diagnosis, patients sought information on TB causes, symptoms, treatment options, designated facilities, costs, complications, and ADRs. However, brochures, online resources, and social media seldom provide timely, personalized, or reliable information.

- Design opportunities: Simple tailored guidance on medications, ADRs, drug resistance, adherence, comorbidities, and treatment progress.

### Treatment adherence

Treatment adherence is a major concern for both patients and healthcare professionals. Patients cited long treatment duration, pill burden, and ADRs, whereas healthcare professionals identified poor adherence among older, male, and uninsured patients. These findings are consistent with previous research that reported symptom guidance as the most common reason for seeking treatment support [33].

- Design opportunities: Personalized reminders, adherence monitoring, and targeted support for high-risk patients.

### Transparency in treatment progress

Patients wanted clearer insight into treatment progress and the rationale for extended therapy, and had a strong psychological need to actively monitor their own adherence and healing journey. The validation and encouragement of patients during exhausting treatment regimens can provide patients with psychological support [34].

- Design opportunities: Visual treatment roadmaps, milestone tracking, and progress updates to support adherence, engagement, and psychological reassurance [35].

### Psychosocial support

In addition to providing clinical support, patients reported substantial emotional and social challenges, including fear of stigma, social isolation, and anxiety related to treatment and contagion, which affected their overall well-being. Previous studies have highlighted the importance of providing patients with psychosocial support and counseling during TB treatment [22,32,36–38].

- Design opportunities: Psychosocial support through empathetic communication, regular well-being check-ins, virtual peer-support resources, and reliable information that reduces misconceptions regarding TB transmission.

### Technological support for TB treatment

Healthcare professionals and patients reported wanting a broader range of digital health functions, including educational resources, referral information, symptom assessment, medication reminders, and appointment scheduling, which are consistent with digital TB intervention recommendations [5,6,14,25,35].

- Design opportunities: TB prevention education integrated with conversational agents, facility information, and SMS reminders to support comprehensive care.

### Treatment affordability

The patient survey suggested a demand for paid services that translate these design opportunities into patient value; however, free provision maximizes accessibility. Even free digital solutions may face structural barriers such as limited phone access, unstable networks, and power limitations [39,40].

- Design opportunities: Sociotechnical systems that empower and facilitate the use of new technologies (e.g., digital literacy education, inclusive digital therapeutics) [41].

### Conversational agents’ potential limitations

Conversational agents cannot replicate genuine empathy and may frustrate patients with their repetitive responses or fail in safety-critical scenarios through context insensitivity, diagnostic hallucinations, or inappropriate crisis triggers [42]. Meanwhile, black-box machine learning models may create accountability gaps for diagnostic errors, while biased training data may perpetuate racial, gender, and linguistic discrimination [42]. Further, text-heavy interfaces can create readability and digital literacy barriers, especially for older or less-educated users [40,43]. These risks are exacerbated by the digital divide, which excludes vulnerable populations from digital health benefits [40].

### Limitations and contributions

This study had several limitations. Convenience sampling, driven by a limited recruitment window, feasibility constraints, and the study’s exploratory nature, may limit the generalizability of our findings. The participants were mostly older adults with stable finances and family support. Therefore, the findings may not reflect more vulnerable groups. Moreover, this study’s Korean-speaking sample restricted its insights into multicultural and multilingual needs. Finally, this study identified user needs and design requirements but did not evaluate the effects of chatbots on treatment adherence and clinical outcomes. Future research should examine user–task–system interactions in both laboratory and real-world environments to evaluate the proposed system’s acceptability and feasibility.

Despite these limitations, the challenges identified in ADR management, treatment adherence, and information are aligned with evidence from other TB settings, suggesting broader relevance for the proposed design considerations [14,18,34]. Although South Korea has a strong IT infrastructure, digital health disparities persist among older adults, who represent a significant proportion of TB patients and often experience declining digital literacy with age [4,14,36–39]. This issue is especially salient in low- and middle-income countries, where rapid smartphone adoption is increasing demand for scalable mHealth solutions [14,18,36,38].

## Conclusions

This study identified the shared challenges experienced by healthcare professionals and patients with TB and translated these findings into practical design considerations for a user-centered conversational agent supporting TB treatment. Healthcare professionals emphasized ADR management, adherence-related challenges, multidrug-resistant TB, and drug-interaction concerns, whereas patients highlighted the need for timely consultations, clear treatment information, psychosocial support, and accessible digital tools. Collectively, these findings suggest that conversational agents for TB treatment should prioritize personalized ADR guidance, understandable educational content, communication support, treatment progress tracking, and usability for older adults. Rather than demonstrating their effectiveness, this study provides a formative evidence base for guiding the design and future evaluation of digital TB support tools in South Korea and similar care settings.

## Supporting information

S1 Fig Chatbot design and features. (TIF)

S2 Fig User interface and core functions of the KakaoTalk-based anti-tuberculosis chatbot. Representative screenshots showing the chatbot workflow, including medication adherence support, tuberculosis education, adverse reaction reporting, healthcare facility information, welfare support resources, and frequently asked question-based patient guidance. The chatbot was designed to improve access to reliable information and support treatment adherence among patients with TB. Original screenshots were translated into English for readability.
(TIF)

S1 Table Healthcare professionals’ perspectives on the barriers to tuberculosis treatment and priorities for digital support (n = 107). (DOCX)

S2 Table Patients’ tuberculosis treatment experiences and preferences for digital health technologies (n = 31). (DOCX)

S3 Table Thematic content analysis of the patient interviews. (DOCX)

## Abbreviations

TB: tuberculosis
ADR: adverse drug reaction
AI: artificial intelligence
DOT: directly observed therapy
mHealth: mobile health
VOT: video-observed therapy
WHO: World Health Organization
