# Supplementary material for: User needs and design opportunities for a conversational agent for tuberculosis treatment: A mixed-methods study
Source: PLOS Digit Health. 2026 Sep 22;5(9):e0001720. doi: 10.1371/journal.pdig.0001720 (PMC13596825; doi:10.1371/journal.pdig.0001720)
Supplement: Supplementary file 1 [file pdig.0001720.s003.docx]

**S1_Table.** Healthcare Professionals’ Perspectives on Challenges in Tuberculosis Treatment and Priorities for Digital Support (n = 107)

| **Responses** | | **n (%)** |
| --- | --- | --- |
| **Challenges in TB treatment*** | |  |
|  | ADR management | 66 (25.1) |
|  | Multidrug resistant TB treatment | 45 (17.1) |
|  | Verification of drug interactions | 41 (15.6) |
|  | Patients’ low treatment adherence | 41 (15.6) |
|  | Lack of patient education time | 28 (10.6) |
|  | Shortage of manpower in public–private mix | 16 (6.1) |
|  | Insufficient financial support for patients | 15 (5.7) |
|  | Lack of educational opportunities for medical staff related to TB | 6 (2.3) |
|  | Others^a^ | 5 (1.9) |
| **Reasons for low medication adherence** | | |
|  | ADRs | 46 (43) |
|  | Lack of self-management skills | 45 (42.1) |
|  | Lack of support from surrounding people | 9 (8.4) |
|  | Others^a^ | 5 (4.7) |
|  | Cognitive impairments such as forgetfulness and dementia | 2 (1.9) |
| **Use of DOT or VOT in TB treatment** | | |
|  | Never use | 82 (76.6) |
|  | Sometimes | 21 (19.6) |
|  | Often | 4 (3.7) |
| **Technological support needed for TB treatment** | | |
|  | Easy access to patients’ medical history | 28 (26.2) |
|  | Rapid diagnosis of TB | 26 (24.3) |
|  | Mobile apps and wearable devices | 23 (21.5) |
|  | Telemedicine approach | 15 (14) |
|  | Big data analysis in healthcare | 10 (9.3) |
|  | None | 5 (4.7) |
| **Digital health technologies needed to assist in TB treatment** | | |
|  | Improvement of medication adherence through automatic notification system | 65 (60.7) |
|  | Quick consultation and emergency understanding via mobile apps | 38 (35.5) |
|  | Securing biometric data through wearable devices | 2 (1.9) |
|  | Others: Smart pill to monitor drug adherence | 2 (1.9) |
| **Required features to be added in AI powered chatbot for TB treatment* (n = 517)** | | |
|  | Medication support | 84 (78.5) |
|  | TB treatment information guide | 60 (56.1) |
|  | Guidance on information about TB | 57 (53.3) |
|  | Communication channel with medical staff | 48 (44.9) |
|  | Screening and management of contacts | 48 (44.9) |
|  | Interface and content considering users with low digital literacy | 45 (42.1) |
|  | Information on TB treatment hospitals and health centers | 45 (42.1) |
|  | Introduction of services for medically underserved communities | 44 (41.1) |
|  | Interconnection with electronic health records | 36 (33.6) |
|  | Data collection for research | 35 (32.7) |
|  | Guidance on symptoms that may indicate TB | 15 (14) |
| **Expected benefits of this chatbot* (n = 517)** | | |
|  | Increased medication adherence | 77 (18.6) |
|  | Improvement of patient safety and convenience during treatment | 70 (16.9) |
|  | Providing accurate information about TB | 59 (14.3) |
|  | Improvement of communication between medical staff and patients | 55 (13.3) |
|  | Economic effects (medical cost savings, time savings in medical treatment, etc.) | 33 (8) |
|  | Improvement of on-site medical treatment efficiency | 28 (6.8) |
|  | Improvement of technology accessibility for older adults with low digital literacy | 24 (5.8) |
|  | Expansion of service benefits for medically underserved communities | 23 (5.6) |
|  | Increased usability for research | 22 (5.3) |
|  | Screening and management of contacts | 22 (5.3) |
| **Expected concerns about this app* (n = 233)** | | |
|  | Low access to information vulnerable groups such as older adults | 81 (75.7) |
|  | Distorted transmission of medical information | 66 (61.7) |
|  | Personal medical information security | 46 (43) |
|  | Regulations of health and medical laws | 23 (21.5) |
|  | Risk of medical accidents | 15 (14) |
|  | Others: Lack of feasibility and service provider’s interest | 2 (1.9) |
| **Desired price for app use (monthly subscription)** | | |
|  | Free | 61 (57) |
|  | KRW 3000 or USD 3/month | 15 (14) |
|  | KRW 5000/ or USD 5/month | 11 (10.3) |
|  | KRW 10000 or USD 10/month | 11 (10.3) |
|  | KRW 100000 or USD 100/month | 9 (8.4) |
| **Requirements for adopting digital technologies for TB treatment* (n = 308)** | | |
|  | Simple and easy to use design for older adults | 80 (26) |
|  | Government support to adopt digital technologies in the medical field | 67 (21.8) |
|  | Support for compensation (fees, subsidies, vouchers, etc.) | 63 (20.5) |
|  | Personal data security configuration/system | 49 (15.9) |
|  | Quick feedback and updates | 49 (15.9) |
| **Likert items** | | Mean (SD) |
|  | Medication adherence of patients in respondent’s clinic | 7.7 (4.1) |
|  | Benefits of using digital health technologies | 6.8 (2.9) |
|  | Willingness to use app | 6.7 (2.5) |
|  | Effectiveness of DOT/VOT in TB treatment in South Korea | 6 (2.4) |

^*^ Multiple responses; ^a^ long-term medication period, difficulty transferring to a TB hospital, consultation room far away from respiratory medicine department, outpatient bankruptcy, long treatment period.

AI: artificial intelligence; PE = Performance Expectancy; EE = Effort Expectancy; SI = Social Influence; FC = Facilitating Conditions; BI = Behavioral Intention; ADR = Adverse Drug Reaction; DOT = Directly Observed Therapy; VOT = Video Observed Therapy.
