# Supplementary material for: User needs and design opportunities for a conversational agent for tuberculosis treatment: A mixed-methods study
Source: PLOS Digit Health. 2026 Sep 22;5(9):e0001720. doi: 10.1371/journal.pdig.0001720 (PMC13596825; doi:10.1371/journal.pdig.0001720)
Supplement: Supplementary file 2 [file pdig.0001720.s004.docx]

**S2_Table.** Patients’ TB treatment experiences and preferences for digital health technologies (n = 31)

| **Patients’ response** | | n (%) |
| --- | --- | --- |
| Type of health facility visited (TB symptoms occurred) | Tertiary hospital | 16 (51.7) |
|  | General hospital | 8 (24.1) |
|  | Clinics | 7 (24.1) |
| TB related education experience | Yes | 29 (93.1) |
|  | Self-study | 1 (3.4) |
|  | Group tutorial | 1 (3.4) |
| Challenges to continuing medication (Multiple responses, n = 80) | Overly long treatment period | 25 (31.3) |
|  | Too many pills | 18 (22.5) |
|  | Severe ADR | 17 (21.3) |
|  | Overly large, difficult to swallow pills | 15 (18.8) |
|  | Doubts about the necessity of taking medication | 3 (3.8) |
|  | Feeling that medication can be stopped owing to improvement in condition | 2 (2.5) |
| Experience of ADRs while on TB medication | Yes | 30 (96.8) |
|  | No | 1 (3.2) |
| ADRs experienced (Multiple responses, n = 74) | Gastrointestinal disorders | 17 (23.2) |
|  | Skin troubles | 15 (20.3) |
|  | Peripheral nerve disorders | 9 (12.2) |
|  | Joint pain | 9 (12.2) |
|  | Visual impairment | 6 (8.1) |
|  | Hepatotoxicity | 6 (8.1) |
|  | Others (loss of appetite, weight loss, shoulder muscle pain, constipation, diarrhea) | 5 (6.8) |
|  | Hematological abnormalities | 4 (5.4) |
|  | Hearing impairment | 2 (2.7) |
|  | Fever | 1 (1.4) |
| Actions taken in response to ADRs* (n = 40) | Visited the clinic/hospital and received prompt action | 16 (40.0) |
|  | Improved on its own without any action | 11 (27.5) |
|  | Received help only by phone | 5 (12.5) |
|  | Acquired information independently through the internet, acquaintances, etc. | 4 (10.0) |
|  | Action taken after receiving an explanation at another hospital | 2 (5.0) |
|  | Unable to act owing to overbooking | 1 (2.5) |
|  | None | 1 (2.5) |
| Channels to acquire information related to TB treatment* (n = 47) | Asking for a doctor during treatment | 26 (55.3) |
|  | Internet search | 12 (25.5) |
|  | Using guidebooks provided by the hospital | 6 (12.8) |
|  | Asking a friend | 1 (2.1) |
|  | Unable to resolve | 1 (2.1) |
|  | Others (none) | 1 (2.1) |
| Demands in the process of diagnosing and treating TB (Multiple responses, n = 79) | Fast consultation related to ADRs | 21 (35) |
|  | Provision of information related to TB | 13 (21.7) |
|  | Guidance and self-diagnosis function for suspected TB symptoms | 12 (20) |
|  | Guidance on TB treatment and public health center information | 8 (13.3) |
|  | Economic support related to TB | 5 (8.3) |
|  | None | 1 (1.7) |
| Priority of the functions in a smartphone app that assists in TB treatment *(multiple responses), n (%) | Guidance on medication side effects | 75 (20.4) |
|  | Guidance on TB self-diagnosis | 48 (13) |
|  | Guide to what TB is | 47 (12.8) |
|  | Guidance on the TB treatment process | 46 (12.5) |
|  | Guidance on TB treatment hospitals and public health centers | 44 (12) |
| Willingness to use the above app | Yes | 19 (62.1) |
|  | No | 12 (37.9) |
| Desired price for using this app (provided at a monthly subscription) | Free | 29 (93.1) |
|  | KRW 1000 or USD 1/month | 1 (3.4) |
|  | KRW 3000 or USD 3/month | 1 (3.4) |

PE = Performance Expectancy; EE = Effort Expectancy; SI = Social Influence; FC = Facilitating Conditions; BI = Behavioral Intention; ADR = Adverse Drug Reaction; DOT = Directly Observed Therapy; VOT = Video Observed Therapy.
