# Supplementary material for: User needs and design opportunities for a conversational agent for tuberculosis treatment: A mixed-methods study
Source: PLOS Digit Health. 2026 Sep 22;5(9):e0001720. doi: 10.1371/journal.pdig.0001720 (PMC13596825; doi:10.1371/journal.pdig.0001720)
Supplement: Supplementary file 3 [file pdig.0001720.s005.docx]

**S3_Table**. Thematic content analysis of the patient interviews

| **Theme** | **Subtheme** | | **Quote** |  |
| --- | --- | --- | --- | --- |
| Medication adherence and related issues | Perceived side effects | *I was just so tired, which was unusual for me. [S04, 35, W]* | | |
|  |  | *Initially, I did not exhibit any signs of TB. I had no phlegm or cough. However, I suddenly started* | | |
|  |  | *coughing a lot in the evening, as if I had allergies, and blood started to come out of my mouth. [S04, 35, W]* | | |
|  |  | *I think I had a skin rash as a side effect. My skin was itchy and warm all over. [S20, 40, W]* | | |
|  |  | *I heard that the side effects include nausea, but the pain is really bad. [S31, 73, W]* | | |
|  |  | *I’m starting to get lazy. I think, “I’m going to get nauseous again if I take this.” [S20, 40, W]* | | |
|  | Perception of drug follow-up | *The doctor told me to take a lower dose of the medication to reduce the side effects. However, even after reducing the dosage, I continued to have negative effects. Thus, I chose to just take the medication for a longer amount of time at a lower dosage. [S20, 40, W]* | | |
|  |  | *I heard that the side effects include nausea. That’s why people don’t finish the treatment, even though it’s a straightforward disease that can be cured with medication. I'm determined to finish the treatment, but I get so nauseous just from taking the medicine. [S31, 73, W]* | | |
|  | Attitude toward non-adherence | *I sometimes eat late, and now the medication is to be taken after meals. So sometimes I forget and take it late. [S20, 40, W]* | | |
|  |  | *I had to take a lot of pills at once, on an empty stomach. It was very uncomfortable. [S08, 69, W]* | | |
|  |  | *I am currently taking a lot of medication that is really overwhelming. [S08, 69, W] In between, there are some pills that have to be taken on an empty stomach. So, if I have to take a* | | |
|  |  | *pill at 5pm, I start to get nervous at 3pm and try not to eat anything. I have to keep an eye on the clock and be careful not to eat anything so I can take the pill on an empty stomach at 5pm. [S04, 35, W]* | | |
|  |  | *I find it very difficult and stressful to take my medication at the correct time. [S04, 35, W]* | | |
|  | Complications due to chronic TB and multidrug regimen | *A few months ago, after switching to the second medication, my liver enzymes skyrocketed. I couldn’t sleep and vomited all night long. I went to the hospital, and the doctor said, “Your liver enzymes are very bad.” So, I was hospitalized. [S04, 35, W]* | | |
|  |  | *I was taking medicine for diabetes at the time. It was probably three years that I had been taking it late. However, my already affected liver got worse when I took the diabetes prescription along with the TB medication. I thus took liver medicine as well. I was so sick of the medicine’s awful taste in food that I was unwilling to eat anything. [S11, 60, M]* | | |
|  |  | *The thing I was most worried about was that TB could lead to lung cancer. I was really scared of that. [S08, 69, W]* | | |
|  |  | *I described my symptoms to my local dermatologist and prescribed medication. [S25, 56, M]* | | |
|  | Healthcare access | *The operating hours are from 9 to 5, so if symptoms occur a bit later than 5 o’clock, I can’t make a call. [S04, 35, W]* | | |
|  |  | *I have encountered numerous side effects. For instance, you can call the counseling center for advice but as I mentioned earlier, I believe it would be beneficial to have an app available during times when the counseling office is closed, allowing you to search for answers. [S04, 35, W]* | | |
|  |  | *Healthcare providers are busy and don't have time to teach you how to take your medication or what to do after you take it. That's the hardest part. [S01, 64, M]* | | |
|  |  | *Firstly, I can contact the TB counseling center by phone, but they are unavailable during the weekends. It was a bit challenging because I couldn't make calls on weekends or on public holidays. [S04, 35, W]* | | |
| Source of information | Asymptomatic or latent TB | *Typically, when someone falls ill with TB, it’s common to test their family members. So, initially, I had a lot of questions about the information related to latent TB. [S04, 35, W]* | | |
|  |  | *If you search, you will find that there are many people who have misconceptions about latent TB and related bacterial matters. These are things that you could easily understand by reading a few pages of a booklet, but there is a lot of incorrect information circulating.* *Latent TB is not something that can be transmitted, yet I’ve come across statements like, “I won’t be around that person because I’m afraid of catching latent TB*.” [S04, 35, W] | | |
|  |  | *It’s absurd to think that I suddenly contracted TB like I did. I believe it’s important for people to have this kind of information. There are numerous asymptomatic cases of TB, so it’s essential to raise awareness that even if you don’t have a cough, phlegm, or other typical symptoms, you could still have it. [S08, 69, W]* | | |
|  | Transmission [Spread of TB] and awareness | *That was my biggest question. How did I transmit this? The doctor told me, “You passed it on to someone else.” I don’t know whom I might have transmitted it to. [S01, 64, M]* | | |
|  |  | *It’s said that 80% of people in my country have latent TB bacteria in their bodies, but I’m pretty healthy, even though poor health can lead to the development of TB. So how did I get it? [S01, 64, M]* | | |
|  |  | *I feel the need for self-diagnosis. Most people don’t know whether they have symptoms or not. I think it’s better to be informed than uninformed, so if someone exhibits certain behaviors or someone, I know it exhibits behaviors that are associated with symptoms, I can give them advice. [S25, 56, M]* | | |
|  |  | *When I was unaware that I was inadvertently causing harm to others, I worked as an elevator operator. … I managed the field and … I interacted with numerous individuals, all of whom had got tested for TB because of me. Therefore, there needs to be an introductory explanation about TB. [S11, 60, M]* | | |
|  | Government welfare service | *It involves a specific calculation, and as a result, the government provides support for things like medication expenses. So, while all serious diseases are concerned, if you happen to contract it because of me, the government offers support, including covering the cost of medication. Therefore, if you do contract it, it’s crucial to know what steps to take promptly and how to address it. [S31, 73, W]* | | |
| Motivators to Adopt technology use | Technology use for timely information | *I’ve heard this a lot since I was in school, and I’m familiar with it. I know that “TB just makes you cough, and later when it gets worse, you bleed and die.” However, if you want to determine whether you have it or not, I wish there was something readily available for that. I hope that both students and adults can easily access it. [S31, 73, W]* | | |
|  |  | *I have encountered numerous side effects. For instance, you can call the counseling center for advice but as I mentioned earlier, I believe it would be beneficial to have an app available during times when the counseling office is closed, allowing you to search for answers. [S04, 35, W]* | | |
|  |  | *I searched the internet mainly for side effects of medications, and the results were easy to understand, even if they weren’t videos or pictures. [S20, 40, W]* | | |
|  |  | *When it comes to TB, I would refer to the information in the booklet and ask my doctor about it. My doctor is usually busy and doesn’t have much time to explain things in detail, so I would just ask questions like, “What is the name of the disease? What medication do I need to take?” At that time, doctors are also busy and have a lot of patients, so they don’t have much time to discuss the disease in depth. [S01, 64, M]* | | |
|  |  | *I didn’t know at that time; I was also very curious about my body. Why did this disease afflict me? In the past, we believed that TB was primarily caused by inadequate nutrition and poverty. However, that’s no longer the prevailing understanding, so I initially had doubts about why I contracted TB. [S01, 64, M]* | | |
|  |  | *I didn’t do an extensive search for information on TB, just what I found on Naver. [S01, 64, M]* | | |
|  |  | *When I first went to the doctor, they gave me some information and gave me a brochure, and I learned a little bit from reading the brochure, but I did a lot of Googling and I didn’t find a lot of additional information, because it was all there in the brochure.* *[S04, 35, W]* | | |
|  | Remote medical service approach or reference to doctor | *Healthcare providers are busy and don’t have time to teach you how to take your medication or what to do after you take it. That’s the hardest part.* *[S01, 64, M] Firstly, I can contact the TB counseling center by phone, but they are unavailable during the weekends. It was a bit challenging because I couldn’t make calls on weekends or on public holidays*. *[S04, 35, W]*  *I’m not sure how helpful the related apps will be. In my opinion, the most important thing is to take the medication as prescribed by the hospital, no matter what. [S01, 64, M] -No, I received a call from the public health center again. I have this issue, and my eyesight is going to worsen slightly. So, I asked, “How can I take medication that harms eyesight and causes side effects?” and they explained that it’s part of a world-class program. Simultaneously, there are individuals who don't experience side effects, but the percentage of those who do is higher. [S08, 69, W]  I was seriously unwell owing to TB medication, and my doctor told me that he had never seen such a serious side effect before, so I felt the need to address the information gap. [S20, 40, W]* | | |
|  | Reminders | *It would be nice if I received a text message. Because, you know, the app can also send a message to my caregiver, like “Hey, did you take your medicine?” That would be great. [S13, 54, M] I constantly forget and need reminders for medication and healthcare. [S20, 40, W]* | | |
| Digital health device use and acceptance | Willingness to pay | *I don’t believe there’s much need for additional costs because you can obtain all the necessary information by consulting the hospital (for free). [S20, 40, W]*  *I believe the government provides financial assistance for one year, as far as I know. A year has passed, and after three months*, *I returned to the hospital, and I’m still receiving financial support. They mentioned that the grace period is only for one year. I asked, and they confirmed it. [S01, 64, M]* | | |
| Perceived social and psychological support | Stigma, isolation, and need for emotional/family support during treatment | *I felt hesitant to contact others even though I was told I wasn’t contagious after a few weeks. [S20, 40, W]*  *At first, I couldn’t even take care of myself. My family helped with everything cooking, cleaning, even reminding me to take my medicine. Without them, I don’t think I could’ve continued the treatment.* *[S08, 69, W]* | | |
