# Supplementary material for: User needs and design opportunities for a conversational agent for tuberculosis treatment: A mixed-methods study
Source: PLOS Digit Health. 2026 Sep 22;5(9):e0001720. doi: 10.1371/journal.pdig.0001720 (PMC13596825; doi:10.1371/journal.pdig.0001720)
Supplement: Supplementary file 4 [file pdig.0001720.s006.docx]

**Comments to Authors**

Thank you for addressing the important and timely issue of tuberculosis (TB) treatment adherence and exploring the role of chatbot-based digital interventions for infectious disease management in this manuscript titled **“Designing a Patient-Centered Chatbot to Enhance Tuberculosis Treatment Adherence: A Mixed-Methods Study.”** The study addresses an important and timely topic, integrating digital health and tuberculosis care through a user-centered design approach. The mixed-methods design and inclusion of both healthcare professionals and patients are commendable and provide meaningful insights for developing scalable digital interventions.

The manuscript is generally well-structured and clearly written. However, several minor revisions are recommended to improve clarity, methodological transparency, and overall presentation.

**Major Strengths**

- The study addresses a relevant global health challenge—TB treatment adherence—using innovative digital solutions.
- The mixed-methods approach enhances the depth and applicability of findings.
- Inclusion of both provider and patient perspectives strengthens the design implications.
- The findings are well-aligned with patient-centered care frameworks and digital health strategies.

**Minor Comments**

**1. Title and Terminology**

- There is a typographical error in the short title (“Patient-Cantered” should be “Patient-Centered”).
- Ensure consistent terminology throughout the manuscript (e.g., “anti-TB chatbot” vs. “TB chatbot”).

**2. Abstract**

- The abstract would benefit from clearer differentiation between healthcare professional and patient samples.
- Consider including additional patient-related quantitative findings.
- Add a brief statement on practical or policy implications to strengthen the conclusion.

**3. Introduction**

- The research gap should be more explicitly articulated, particularly distinguishing this study from prior chatbot or mHealth adherence studies.
- Include more recent references (e.g., 2023–2025) on AI/chatbots in TB or digital health interventions.

**4. Methods**

- Clarify inclusion and exclusion criteria for patient recruitment.
- Indicate whether a sample size justification or power consideration was conducted.
- Provide more details on:
  - Analysis of Likert-scale data
  - Inter-rater reliability (e.g., specify statistics such as Cohen’s kappa)
- Minor correction: “Likertscale” should be “Likert scale.”

**5. Results**

- Consider reporting variability measures (e.g., standard deviation or confidence intervals) where applicable.
- Reduce redundancy between text and figures.
- Clearly distinguish between survey findings and interview-derived themes.

**6. Figures and Tables**

- Improve clarity of Figures (especially Figures 2 and 3); axis labels and percentage scales are not fully clear.
- Enhance figure captions with more descriptive explanations.
- Ensure consistent formatting across tables (alignment, percentage reporting).

**7. Discussion**

- Some sections repeat results; consider streamlining to enhance readability.
- Strengthen comparison with existing literature on chatbot-based or digital adherence interventions.
- Briefly discuss potential limitations of AI/chatbots (e.g., misinformation risk, digital literacy barriers).

8.3 **Language and Formatting**

- Address minor grammatical issues (e.g., “participation qualitative study” → “participation in the qualitative study”).
- Ensure consistent tense usage and use of Standardize abbreviations
