# Supplementary material for: User needs and design opportunities for a conversational agent for tuberculosis treatment: A mixed-methods study
Source: PLOS Digit Health. 2026 Sep 22;5(9):e0001720. doi: 10.1371/journal.pdig.0001720 (PMC13596825; doi:10.1371/journal.pdig.0001720)
Supplement: Supplementary file 5 [file pdig.0001720.s007.docx]

**Response letter**

Dear Editor and Reviewers,

We sincerely thank you for the time and effort invested in reviewing our manuscript, "Designing a Patient-Centered Chatbot to Enhance Tuberculosis Treatment Adherence: A Mixed-Methods Study." We greatly appreciate the constructive comments and suggestions, which have substantially improved the quality, clarity, and rigor of the manuscript.

We have carefully revised the manuscript in response to all comments and revised content are highlighted in blue color throughout the manuscript.

**#1- Responses to the journal office requirements**

**Comment 1.** Please amend the detailed Financial Disclosure statement in full sentences, including grants, grant numbers, organizations, author initials, funder roles, and salary support.

**Response:** We thank the journal for this guidance. We have revised the Financial Disclosure statement into full publishable sentences and included the funding organization, grant number, recipient author initials, and funder role. We have also clarified whether any authors received salary support from the funder. [Page 20, Line 412-414]

Revised Financial Disclosure statement:
This study was supported by the Severance Hospital Research Fund for Clinical Excellence (grant number: C-2022-0017 to YP). The funders had no role in the study design, data collection and analysis, decision to publish, or manuscript preparation of the manuscript

.

**Comment 2.** PLOS requires that all data underlying the findings be freely available unless an ethics/privacy exemption is approved.

**Response:** We appreciate this important policy reminder. Because this study involves human participants and potentially identifiable qualitative interview data from patients receiving TB treatment, unrestricted public release of the full qualitative dataset may compromise participant confidentiality and may exceed the scope of the original ethics approval. We have therefore revised the Data Availability Statement and will separately provide a formal exemption explanation to the journal office. [Page 20, Line 419-422]

**Comment 3**.

Please address possible copyright issues, especially the graphical abstract and Figure 1 screenshots.

**Response:** We have addressed these concerns as follows:

- Graphical abstract: We reviewed all visual elements and confirmed which were author-created. Any third-party icons/images have been removed and replaced with original or openly licensed alternatives compatible with CC BY 4.0, with appropriate attribution where required. [Page 20, Line 415-417]
- Fig 1 and supplement figure screenshots: We understand that screenshots of copyrighted platform interfaces may not be suitable for publication under CC BY 4.0. Accordingly, we have replaced or redrawn Figure 1 as an original schematic/mock interface illustration showing the chatbot flow and functions without reproducing copyrighted interface elements or browser/app controls. We will provide any additional licensing details requested by the journal office. [Page 7-8 and supplementary file]

**Comment 4:** Please provide an Author Summary of 150-200 words.

**Response:** We have added an Author Summary between the Abstract and Introduction.

Author Summary (about 198 words):
Tuberculosis (TB) treatment can be difficult to complete because it is prolonged and often involves side effects, uncertainty, stigma, and limited opportunities to ask questions during clinic visits. Digital tools such as conversational agents can help patients manage treatment; however, few studies have examined what patients and healthcare professionals need from such tools in TB care. In this mixed-methods study, we surveyed 107 healthcare professionals and 31 patients with TB and interviewed 10 patients in South Korea to identify barriers to use, unmet needs, and design priorities for a user-centered conversational agent supporting TB care (hereafter, anti-TB conversational agent). Healthcare professionals highlighted ADRs, adherence-related challenges, multidrug-resistant TB, and drug interaction management as major concerns. Patients emphasized the need for rapid guidance regarding side effects, clearer TB information, easier communication with providers, and emotional support during treatment. Patients preferred solutions that were user-friendly, affordable, and integrated into familiar platforms. Accordingly, we identified key design priorities, including early detection and TB awareness, timely and customized information access, personalized support for treatment adherence, transparency in treatment progress, psychosocial and technological support, and treatment affordability. These findings can guide the development and future evaluation of user-centered conversational agents for TB treatment-adherence support. [Page 4. Line 56-73]

**Comment 5:** Table 3 is cited but not included.

**Response**: We have corrected and rearranged the table numbers in order, by ensuring that Table 4 (revised no, thematic content analysis of patient interviews) is included in the manuscript and cited consistently in the text. We also checked that all other tables and supplementary materials are properly numbered and cited. [Page 13, Line 270; Table 4 in page 31]

**#2: RESPONSE TO ADDITIONAL EDITOR COMMENTS**

 SE: Both reviewers agreed on the value of the manuscript. However, there are also major points to consider. In addition to the comments by the reviewers, please make sure to address the following items:

**Responses to the Senior Editor**

We fully agree and have revised the manuscript accordingly. The revised title, abstract, discussion, and conclusion no longer claim that the chatbot enhances adherence or demonstrates efficacy. Instead, the manuscript is now presented as a mixed-methods needs-assessment and formative design study that identifies user needs, barriers, and design opportunities for a TB treatment-support chatbot.

**Comment 1.** Reframe the manuscript as a formative design/needs-assessment study unless actual chatbot use and evaluation data are available

Response: We fully agree and have revised the manuscript accordingly. The revised title, abstract, discussion, and conclusion no longer claim that the chatbot enhances adherence or demonstrates efficacy. Instead, the manuscript is now presented as a mixed-methods needs-assessment and formative design study that identifies user needs, barriers, and design opportunities for a TB treatment-support chatbot. [**Title, abstract**: Page 1-3; **Discussion**: Page 15-18]

**Comment 2.** Revise title, abstract, and conclusion to remove unsupported claims of adherence improvement or efficacy

Response: Completed. We carefully removed or softened unsupported causal language throughout the manuscript. [**Title**: page 1, Line 1-4; **Abstract:** Page 2, Line 23-35, 46-50].

**Comment 3.** Provide full details of recruitment, eligibility, response rates, sampling method, and sample size rationale

Response: We expanded the Methods section to provide: A statement clarifying that this was an exploratory formative study and that no formal power calculation was performed

Suggested wording for Methods:
Participants were recruited from tertiary referral hospitals, general hospitals, clinics, and public health centers involved in TB care. Eligible patient participants included physicians and nurses engaged in TB diagnosis, treatment, counseling, or case management. Eligible participants were adults receiving TB treatment at Severance Hospital who had taken anti-TB medication for at least three months and were able to provide informed consent. Patients with severe cognitive or communication difficulties that precluded participation were excluded. This study was designed as an exploratory, formative, needs-assessment study; therefore, no formal sample size calculation was performed. [Page 8-Line 157-164]

**Comment 4.** Double check all tables, denominators, percentages, scales, and missing data reporting

Response: We carefully reviewed all tables including numbers, percentages, denominators, and scales and corrected inconsistencies where identified. We also clarified missing responses where applicable and ensured that percentages are based on the appropriate denominator, including multiple-response items. [Table 1-4, Page 27-31]

**Comment 5.** Include the survey instruments as appendix

Response: Completed. We added the healthcare professional survey and patient survey instruments as supplementary appendices and cited them in the Methods section.

**Comment 6.** Strengthen qualitative reporting, e.g., use COREQ, and report saturation, coding procedures, and inter-rater reliability where applicable

**Response:** We strengthened the qualitative reporting in accordance with the COREQ guidelines. The revised manuscript provides additional details on transcript management, NVivo™-assisted inductive content analysis, open coding, category and theme development, and data saturation. We also clarified that two researchers independently coded the transcripts and resolved coding discrepancies through discussion and consensus. Because no formal inter-rater reliability statistics were calculated, we did not report one. [Page 11, Line 217-220]

**Comment 7.** Clarify the chatbot architecture, content source, AI/rule-based status, clinical governance, and privacy/security aspects

**Response:** Thank you for this valuable suggestion. We have substantially expanded the chatbot description in the Methods section.

Specifically, we now describe the chatbot platform and architecture, clarify that the system used a rule-based conversational structure rather than generative AI, identify the sources of educational and clinical content, explain the role of clinical experts in content development and review, and provide additional information regarding privacy protection and data management procedures. These additions improve transparency regarding the chatbot's development, governance, and intended role as a patient support tool. [Page 7-8, Line 133-147]

-----------------------------------------------------------

**#3: Responses to the Associate Editor comments**

Thank you for your valuable contribution to an important research topic.
Because we had trouble recruiting sufficient reviewers, I reviewed the paper. My comments are given below. They are few, because the paper is fundamentally strong.
Please attend carefully to Reviewer 1's insightful comments - addressing them will certainly improve the paper. I also believe that tending to my comments (or one main comment) below will yield a higher impact paper.
Thank you, Charles Delahunt

**Response:** We appreciate this valuable suggestion and revised the Discussion to improve readability and actionability. We now present the key design opportunities more explicitly with subheadings and concise synthesis linked to supporting findings. We also reduced repetitive summary text to improve density and flow.

The following revisions are made as per your suggestions:

- Line numbers throughout the manuscript to make an easier review
- Added clearer substructure to the Discussion
- Consolidated repetitive paragraphs
- Highlighted actionable design implications
- Improved linkage between findings and design recommendations

**Comment 1**. Line numbers make reviews much easier

**Response:** Thank you for the valuable comment, we have included the line numbers throughout the manuscript as suggested.

**Main point:**
**Comment 2**. Format of the Discussion: the conversational format (paragraphs of text) tends to blur the various findings, making it hard to extract the key actionable items along with their key supporting datapoints.

**Response:** Thank you for this valuable suggestion. We have substantially reorganized the Discussion section around the TB treatment journey and grouped findings into thematic categories. Key findings are now linked directly to corresponding design opportunities, making the actionable implications easier to identify and interpret. [Page 15-18]

**Comment 3.** Formatting: make "key design opportunities" jump out (eg bold font heading; separate heading with bullet points, etc), so that a reader can quickly find the actionable content.

**Response:** We agree. The Discussion has been reformatted to improve readability. Key design opportunities are now presented under distinct subheadings and highlighted separately from the supporting findings. This structure allows readers to quickly identify practical recommendations for chatbot development. [Page 15-18]

**Comment 4.** Is it possible to reduce word count to increase density (to enable the reader to more efficiently extract the actionable content)?

**Response:** Thank you for this recommendation. We carefully revised the Discussion to reduce redundancy, improve conciseness, and increase the density of key messages. Repetitive descriptions were removed, and related findings were consolidated while preserving important supporting evidence and references.

*Total word counts have been reduced to ‘3379’ (introduction to conclusion)*

**Comment 5.** Perhaps you can make a draft that incorporates the above options, then test-drive it on some colleagues unacquainted with the work.

**Response:** We appreciate this thoughtful suggestion. Following the revision, the Discussion was reviewed by colleagues who were not directly involved in the study to assess clarity, organization, and readability. Their feedback informed further refinements to the structure and presentation of the key design opportunities.

**Minor points:
Comment 6.** "Healthcare professionals still identified adherence as the area most in need of technological support”: this appears to contradict the earlier listing, "the main challenges in TB treatment included ..."

**Response:** Thank you for pointing this out. While adverse drug reactions and multidrug-resistant TB were reported as major treatment challenges, healthcare professionals most frequently identified treatment adherence as the area where digital technologies could provide the greatest support (Survey question 15).

**Comment 7.** "Healthcare professionals expressed the need for various forms of technological support in managing TB treatment." : Are health care providers an audience for this chatbot? I thought not, but this makes it sound like they are.

**Response:** We appreciate this observation. The chatbot was developed to mediate clinician-patient communication and provide features for both clinicians and patients. While patients are primary users, clinicians access and utilize patient-generated data. Therefore, it was important to understand the demands of both parties, which is why we collected data from both clinicians and patients.

**Trivial points:**
**Comme**nt 8. "Research shows users’ specific needs and challenges to develop culturally sensitive conversational agents”: unclear clause

**Response:** Thank you. The sentence has been revised for clarity and now reads: “Previous research highlights that, when developing culturally sensitive conversational agents, understanding users’ needs and challenges is important to ensure their relevance and effectiveness across diverse patient populations worldwide.” [Page 6; line 106-108; page 19; Line 378-381]

**Comment 9.** "Many found existing materials too basic and lacked tailored content." : grammar

**Response:** Thank you for identifying this issue. The sentence and the whole manuscript have been revised English and grammar.

**Comment 10.** "As our empirical data were collected at a tertiary hospital in South Korea, regional and cultural biases may exist and limit the generalizability of our findings. Our findings are based on data collected from a tertiary hospital in South Korea, where patients ..." : Repetitive

**Response:** Thank you and we revised the content as: the repetitive statements have been consolidated into a single paragraph in the Limitations section to improve readability and avoid redundancy. [Page 16 – Limitations]

-----------------------------------------------------------

**# 4. Response to reviewer 1**

**Comments to Authors**

Thank you for addressing the important and timely issue of tuberculosis (TB) treatment adherence and exploring the role of chatbot-based digital interventions for infectious disease management in this manuscript titled “Designing a Patient-Centered Chatbot to Enhance Tuberculosis Treatment Adherence: A Mixed-Methods Study.” The study addresses an important and timely topic, integrating digital health and tuberculosis care through a user-centered design approach. The mixed-methods design and inclusion of both healthcare professionals and patients are commendable and provide meaningful insights for developing scalable digital interventions. The manuscript is generally well-structured and clearly written. However, several minor revisions are recommended to improve clarity, methodological transparency, and overall presentation.

**Major Strengths**

**Comment 1:** The study addresses a relevant global health challenge—TB treatment adherence—using innovative digital solutions. The mixed-methods approach enhances the depth and applicability of findings. Inclusion of both provider and patient perspectives strengthens the design implications. The findings are well-aligned with patient-centered care frameworks and digital health strategies.

**Response:**

We sincerely thank you for the time and effort invested in reviewing our manuscript, "Designing a Patient-Centered Chatbot to Enhance Tuberculosis Treatment Adherence: A Mixed-Methods Study." We greatly appreciate the constructive comments and suggestions, which have substantially improved the quality, clarity, and rigor of the manuscript.

We have carefully revised the manuscript in response to all comments and revised content are highlighted in blue color. The point-to-point responses are as follows:

**Minor Comments**

**Comment 2. Title and Terminology**

- There is a typographical error in the short title (“Patient-Cantered” should be “Patient-Centered”). Ensure consistent terminology throughout the manuscript (e.g., “anti-TB chatbot” vs. “TB chatbot”).

**Response:** All terminology has been standardized throughout the manuscript, including consistent use of "anti-TB chatbot" and correction of typographical errors.

**Comment 2. Abstract:** The abstract would benefit from clearer differentiation between healthcare professional and patient samples. Consider including additional patient-related quantitative findings. Add a brief statement on practical or policy implications to strengthen the conclusion.

**Response**: The abstract was revised to clearly distinguish healthcare professional and patient findings and to include additional patient-centered results and practical implications [Page 2-3; Line 23-50].

**Comment 3. Introduction**

- The research gap should be more explicitly articulated, particularly distinguishing this study from prior chatbot or mHealth adherence studies.
- Include more recent references (e.g., 2023–2025) on AI/chatbots in TB or digital health interventions.

**Response:** We strengthened the research gap by distinguishing this study from prior work on general mHealth adherence tools and chatbot research not grounded in TB-specific user needs. We also updated the introduction and discussion with more recent literature where appropriate, especially on digital health implementation, chatbot design, and TB adherence support. [Page 5-7; Line 94-108, 105-113, 116-130]

**Comment 4. Methods:** Clarify inclusion and exclusion criteria for patient recruitment. Indicate whether a sample size justification or power consideration was conducted. Provide more details on: Analysis of Likert-scale data, Inter-rater reliability correction: “Likertscale” should be “Likert scale.”

**Response:** Thank you for this suggestion. We have expanded the Methods section to provide additional details regarding the patient survey. We have clarified our sampling methods and conducted a sample size justification where necessary. The revised manuscript now describes the domains covered by the questionnaire, including treatment experiences, medication adherence challenges, adverse drug reactions, information-seeking behaviors, unmet support needs, preferred digital health features, willingness to use a TB chatbot, and acceptable subscription costs. We also clarified the reliability of the survey instrument and intercoder reliability of qualitative data analysis. [Page 9; Line 167-182]

**Comment 5. Results:** Consider reporting variability measures (e.g., standard deviation or confidence intervals) where applicable. Reduce redundancy between text and figures. Clearly distinguish between survey findings and interview-derived themes.

**Response:** Thank you for these suggestions. We have revised the Results section to improve clarity and reporting. Redundant descriptions that duplicated information already presented in figures and tables were removed. In addition, the Results section has been reorganized to clearly separate quantitative survey findings from qualitative interview findings, with thematic analysis results presented under dedicated subheadings.

**Comment 6. Figures and Tables:** Improve clarity of Figures (especially Figures 2 and 3); axis labels and percentage scales are not fully clear. Enhance figure captions with more descriptive explanations. Ensure consistent formatting across tables (alignment, percentage reporting)

**Response:** Thank you for these helpful comments. Figures 2 and 3 have been revised to improve readability, including clearer axis labels, percentage scales, and graphical formatting. Figure captions have been expanded to provide more detailed explanations of the data presented. In addition, all tables were reviewed and reformatted to ensure consistency in alignment, decimal places, percentage reporting, and presentation style throughout the manuscript.

**Comment 7. Discussion:** Some sections repeat results; consider streamlining to enhance readability. Strengthen comparison with existing literature on chatbot-based or digital adherence interventions. Briefly discuss potential limitations of AI/chatbots (e.g., misinformation risk, digital literacy barriers).

**Response:** We appreciate these suggestions. The Discussion section has been substantially revised to reduce repetition and improve readability. We reorganized the discussion around the TB treatment journey and highlighted key design opportunities under separate thematic sections. Additional comparisons with recent studies on chatbot-based and digital adherence interventions were incorporated to better contextualize our findings. We also expanded the limitations and implementation considerations to discuss challenges related to digital literacy, misinformation, accessibility among older adults, privacy concerns, and the limitations of chatbot-based support in clinical settings. [Page 15-18]

**Comment 8**. **Language and Formatting:** Address minor grammatical issues (e.g., “participation qualitative study” → “participation in the qualitative study”). Ensure consistent tense usage and use of Standardize abbreviations

**Response:** Thank you for identifying these issues. The manuscript has undergone comprehensive language editing to improve grammar, readability, and consistency. Identified grammatical errors were corrected, terminology was standardized throughout the manuscript, abbreviations were defined at first use and used consistently thereafter, and verb tense usage was reviewed to ensure consistency across all sections. [Editage report]

-----------------------------------------------------------
