# Supplementary material for: User needs and design opportunities for a conversational agent for tuberculosis treatment: A mixed-methods study
Source: PLOS Digit Health. 2026 Sep 22;5(9):e0001720. doi: 10.1371/journal.pdig.0001720 (PMC13596825; doi:10.1371/journal.pdig.0001720)
Supplement: Peer review history

# Peer review: Decision Letter 0

**DOI:** 10.1371/journal.pdig.0001720.r001

**Authors:** Charles B. Delahunt, Baki Kocaballi

9 Jun 2026

Response to Reviewers Revised Manuscript with Track Changes Manuscript **Journal Requirements:**

1. Please amend your detailed Financial Disclosure statement. This is published with the article. It must therefore be completed in full sentences and contain the exact wording you wish to be published.

i. Please clarify all sources of financial support for your study. List the grants, grant numbers, and organizations that funded your study, including funding received from your institution. Please note that suppliers of material support, including research materials, should be recognized in the Acknowledgements section rather than in the Financial Disclosure.

ii. State the initials, alongside each funding source, of each author to receive each grant. For example: "This work was supported by the National Institutes of Health (####### to AM; ###### to CJ) and the National Science Foundation (###### to AM)."

iii. State what role the funders took in the study. If the funders had no role in your study, please state: “The funders had no role in study design, data collection and analysis, decision to publish, or preparation of the manuscript.”

iv. If any authors received a salary from any of your funders, please state which authors and which funders.

2. In the online submission form, you indicated that “The datasets generated and/or analyzed during the current study are available from the corresponding author upon reasonable request. As this research is part of an ongoing project involving clinical data, some restrictions apply, and the full dataset is not publicly available at this stage to protect participant confidentiality and comply with ethical regulations.”.

All PLOS journals now require all data underlying the findings described in their manuscript to be freely available to other researchers, either

1. In a public repository,

2. Within the manuscript itself, or

3. Uploaded as supplementary information.

This policy applies to all data except where public deposition would breach compliance with the protocol approved by your research ethics board. If your data cannot be made publicly available for ethical or legal reasons (e.g., public availability would compromise patient privacy), please explain your reasons by return email and your exemption request will be escalated to the editor for approval. Your exemption request will be handled independently and will not hold up the peer review process, but will need to be resolved should your manuscript be accepted for publication. One of the Editorial team will then be in touch if there are any issues.

3. Some material included in your submission may be copyrighted. According to PLOS’s copyright policy, authors who use figures or other material (e.g., graphics, clipart, maps) from another author or copyright holder must demonstrate or obtain permission to publish this material under the Creative Commons Attribution 4.0 International (CC BY 4.0) License used by PLOS journals. Please closely review the details of PLOS’s copyright requirements here: PLOS Licenses and Copyright. If you need to request permissions from a copyright holder, you may use PLOS's Copyright Content Permission form.

Please respond directly to this email or email the journal office and provide any known details concerning your material's license terms and permissions required for reuse, even if you have not yet obtained copyright permissions or are unsure of your material's copyright compatibility.

Potential Copyright Issues:

a. Graphical Abstract: Please confirm whether you drew the images / clip-art within the figure panels by hand. If you did not draw the images, please provide (a) a link to the source of the images or icons and their license / terms of use; or (b) written permission from the copyright holder to publish the images or icons under our CC-BY 4.0 license. Alternatively, you may replace the images with open source alternatives. See these open source resources you may use to replace images / clip-art:

- https://commons.wikimedia.org

- https://openclipart.org/

b. Figure 1 contains screenshots. We are not permitted to publish these under our CC-BY 4.0 license; websites are usually intellectual property and are copyrighted. This includes peripheral graphics of the web browser such as the [X] buttons. We ask that you please remove or replace it.

4. Please provide an Author Summary. This should appear in your manuscript between the Abstract (if applicable) and the Introduction, and should be 150–200 words long. The aim should be to make your findings accessible to a wide audience that includes both scientists and non-scientists. Sample summaries can be found on our website under Submission Guidelines:

https://journals.plos.org/digitalhealth/s/submission-guidelines#loc-parts-of-a-submission

5. We have noticed that you have cited Table 3 in the manuscript file but there are no corresponding tables in the manuscript. Please amend your manuscript to include this table, noting that tables should not be uploaded as individual files.

**Additional Editor Comments (if provided):**

Both reviewers agreed on the value of the manuscript. However, there are also major points to consider. In addition to the comments by the reviewers, please make sure to address the following items:

- Reframe the manuscript as a formative design/needs-assessment study unless actual chatbot use and evaluation data are available. The study does not demonstrate that the chatbot enhances adherence. It identifies needs and design opportunities.

- Revise the title, abstract, and conclusion to remove unsupported claims of adherence improvement or efficacy.

- Provide full details of recruitment, eligibility, response rates, sampling method, and sample size rationale.

- Double check the info on all tables, denominators, percentages, scales, and missing-data reporting.

- Include the survey instruments as appendix

- Strengthen qualitative reporting. eg use COREQ, and report saturation, coding procedures, and inter-rater reliability where applicable.

- Clarify the chatbot architecture, content source, AI/rule-based status, clinical governance, and privacy/security aspects.

-----------------------------------------------------------

AE:

Thank you for a valuable contribution to an important research topic.

Because we had trouble recruiting sufficient reviewers, I reviewed the paper. My comments are given below. They are few, because the paper is fundamentally strong.

Please attend carefully to Reviewer 1's insightful comments - addressing them will certainly improve the paper. I also believe that tending to my comments (or one main comment) below will yield a higher impact paper.

Thank you, Charles Delahunt

-----------------------------------------------------------

Line numbers make review much easier

Main point:

Format of the Discussion: the conversational format (paragraphs of text) tends to blur the various findings, making it hard to extract the key actionable items along with their key supporting datapoints.

Formatting: make "key design opportunities" jump out (eg bold font heading; separate heading with bullet points, etc), so that a reader can quickly find the actionable content.

Is it possible to reduce word count to increase density (to enable the reader to more efficiently extract the actionable content)?

Perhaps you can make a draft that incorporates the above options, then test-drive it on some colleagues unacquainted with the work.

----------------------------------------------

Minor points:

"healthcare professionals still identified adherence as the area most in need of technological

support " : this appears to contradict the earlier listing, "the main challenges in TB treatment included ..."

" healthcare professionals expressed the need for various forms of technological support in managing TB treatment." : Are health care providers an audience for this chatbot?. I thought not, but this makes it sound like they are.

-------------------------------------------------------

Trivial points:

"Research shows users’ specific needs and challenges to develop culturally sensitive conversational agents" : unclear clause

"Many found existing materials too basic and lacked tailored content." : grammar

**Reviewers' Comments:**

**Comments to the Author**

1. Does this manuscript meet PLOS Digital Health’s publication criteria?

Reviewer #1: Yes

**********

2. Has the statistical analysis been performed appropriately and rigorously?

Reviewer #1: Yes

**********

3. Have the authors made all data underlying the findings in their manuscript fully available (please refer to the Data Availability Statement at the start of the manuscript PDF file)?

The PLOS Data policy

Reviewer #1: No

**********

4. Is the manuscript presented in an intelligible fashion and written in standard English?

Reviewer #1: Yes

**********

**Reviewer #1:**  The study is well-structured and makes a valuable contribution by integrating a mixed-methods design and incorporating perspectives from both healthcare professionals and patients, which strengthens the relevance and applicability of the findings. However, several minor revisions are recommended to further improve clarity and rigor. Specifically, the manuscript would benefit from clearer articulation of the research gap in the introduction, inclusion of more recent literature on chatbot-based interventions, and additional methodological details such as participant selection criteria and inter-rater reliability. The abstract could be refined to better distinguish between study populations and highlight key findings. Minor improvements are also suggested for figures and tables to enhance clarity, as well as for language consistency and grammatical accuracy throughout the manuscript. Addressing these points will strengthen the overall quality and readability of the study.

**********

what does this mean?). If published, this will include your full peer review and any attached files.

**Do you want your identity to be public for this peer review?**  If you choose “no”, your identity will remain anonymous but your review may still be made public.

For information about this choice, including consent withdrawal, please see our Privacy Policy

Reviewer #1: No

**********

**Figure resubmission:**

**Reproducibility:**

To enhance the reproducibility of your results, we recommend that authors of applicable studies deposit laboratory protocols in protocols.io, where a protocol can be assigned its own identifier (DOI) such that it can be cited independently in the future. Additionally, PLOS ONE offers an option to publish peer-reviewed clinical study protocols. Read more information on sharing protocols at https://plos.org/protocols?utm_medium=editorial-email&utm_source=authorletters&utm_campaign=protocols

Attachment Submitted filename: Comments to Authors_PDGI-D-26-00033.docx

# Author comment: Author response to Decision Letter 1

**DOI:** 10.1371/journal.pdig.0001720.r002

16 Aug 2026

Attachment Submitted filename: Response to all the comments_Clean_Final.docx

# Editor report: Decision Letter 1

**DOI:** 10.1371/journal.pdig.0001720.r003

**Authors:** Charles B. Delahunt, Baki Kocaballi

25 Aug 2026

User needs and design opportunities for a conversational agent for tuberculosis treatment: A mixed-methods study

PDIG-D-26-00033R1

Dear Dr. Rajaguru,

We are pleased to inform you that your manuscript 'User needs and design opportunities for a conversational agent for tuberculosis treatment: A mixed-methods study' has been provisionally accepted for publication in PLOS Digital Health.

Before your manuscript can be formally accepted you will need to complete some formatting changes, which you will receive in a follow-up email from a member of our team.

Please note that your manuscript will not be scheduled for publication until you have made the required changes, so a swift response is appreciated.

IMPORTANT: The editorial review process is now complete. PLOS will only permit corrections to spelling, formatting or significant scientific errors from this point onwards. Requests for major changes, or any which affect the scientific understanding of your work, will cause delays to the publication date of your manuscript.

If your institution or institutions have a press office, please notify them about your upcoming paper to help maximize its impact. If they'll be preparing press materials, please inform our press team as soon as possible -- no later than 48 hours after receiving the formal acceptance. Your manuscript will remain under strict press embargo until 2 pm Eastern Time on the date of publication. For more information, please contact digitalhealth@plos.org.

Thank you again for supporting Open Access publishing; we are looking forward to publishing your work in PLOS Digital Health.

Best regards,

Charles B. Delahunt

Section Editor

PLOS Digital Health

***********************************************************

**Additional Editor Comments (if provided):**

Thank you for your attention to the various reviewers' comments, and for an interesting and valuable piece of research.

**Reviewer Comments (if any, and for reference):**
